# Doxorubicin Regulates Autophagy Signals via Accumulation of Cytosolic Ca^2+^ in Human Cardiac Progenitor Cells

**DOI:** 10.3390/ijms17101680

**Published:** 2016-10-09

**Authors:** Ji Hye Park, Sung Hyun Choi, Hyungtae Kim, Seung Taek Ji, Woong Bi Jang, Jae Ho Kim, Sang Hong Baek, Sang Mo Kwon

**Affiliations:** 1 Laboratory of Regenerative Medicine and Stem Cell Biology, Department of Physiology, Medical Research Institute, School of Medicine, Pusan National University, Yangsan 50612, Korea; siwonvin@naver.com (J.H.P.); jst5396@hanmail.net (S.T.J.); jangwoongbi@naver.com (W.B.J.); 2 Cellular Therapeutics Team, Bio Reseach and Development Center, Daewoong Pham. Co., Ltd., Seoul 06170, Korea; shchoi0704@naver.com; 3 Department of Thoracic and Cardiovascular Surgery; Pusan National University Yangsan Hospital, Yangsan 50612, Korea; 2719k@naver.com; 4 Research Institute of Convergence Biomedical Science and Technology, Pusan National University School of Medicine, Yangsan 50612, Korea; jhkimst@pusan.ac.kr; 5 Division of Cardiology, Seoul St. Mary’s Hospital, School of Medicine, The Catholic University of Korea, Seoul 06591, Korea

**Keywords:** cardiac progenitor cell, autophagy, doxorubicin, rapamycin, cardiotoxicity

## Abstract

Doxorubicin (DOXO) is widely used to treat solid tumors. However, its clinical use is limited by side effects including serious cardiotoxicity due to cardiomyocyte damage. Resident cardiac progenitor cells (hCPCs) act as key regulators of homeostasis in myocardial cells. However, little is known about the function of hCPCs in DOXO-induced cardiotoxicity. In this study, we found that DOXO-mediated hCPC toxicity is closely related to calcium-related autophagy signaling and was significantly attenuated by blocking mTOR signaling in human hCPCs. DOXO induced hCPC apoptosis with reduction of SMP30 (regucalcin) and autophagosome marker LC3, as well as remarkable induction of the autophagy-related markers, Beclin-1, APG7, and P62/SQSTM1 and induction of calcium-related molecules, CaM (Calmodulin) and CaMKII (Calmodulin kinase II). The results of an LC3 puncta assay further indicated that DOXO reduced autophagosome formation via accumulation of cytosolic Ca^2+^. Additionally, DOXO significantly induced mTOR expression in hCPCs, and inhibition of mTOR signaling by rapamycin, a specific inhibitor, rescued DOXO-mediated autophagosome depletion in hCPCs with significant reduction of DOXO-mediated cytosolic Ca^2+^ accumulation in hCPCs, and restored SMP30 and mTOR expression. Thus, DOXO-mediated hCPC toxicity is linked to Ca^2+^-related autophagy signaling, and inhibition of mTOR signaling may provide a cardio-protective effect against DOXO-mediated hCPC toxicity.

## 1. Introduction

Doxorubicin (DOXO) is an anthracycline antibiotic, widely used in cancer chemotherapy [1]. Despite its highly beneficial effects against cancer, the clinical use of DOXO is limited by side effects including serious cardiotoxicity due to the loss of cardiomyocytes [2,3,4]. The intracellular signaling and molecular mechanism of DOXO-induced cardiotoxicity remain controversial. Recent studies have suggested that DOXO-mediated cardiotoxicity may be related to iron-mediated calcium accumulation and oxidative stress [5,6,7], which cause mitochondrial dysfunction [8]. However, the precise mechanisms underlying cardiotoxicity have not been clearly elucidated.

Human cardiac stem/progenitor cells (hCPCs) were first discovered in 2003 by Bernardo Nadal Ginard, Piero Anversan and collegues [9]. Although hCPCs are found in small numbers in the heart, they contribute to the repair of damaged heart tissue via direct multi-lineage differentiation into various types of cells including endothelial cells (ECs), cardiomyocytes (CMs), and smooth muscle cells (SMCs) [10]. Recent studies show that CPCs play a critical role in cardiomyocyte homeostasis [11]. Although studies to date suggest that DOXO-induced cardiotoxicity leads to CSC depletion [12], the underlying mechanisms of DOXO-induced cardiotoxicity have not been fully determined.

Autophagy has emerged as an important intracellular signaling pathway for the regulation of cellular activities including cell proliferation, differentiation, and replicative senescence. It has been reported that dysregulation of autophagy may be closely related to an imbalance in cellular metabolism including mitochondrial dysfunction and cell toxicity [13,14,15]. Recently accumulated data provide a correlation between onsets of autophagy and doxorubicin-mediated cardiotoxicity [15,16,17]. Decuypere et al. reported that DOXO-induced cardiotoxicity is closely related to malfunction of autophagy, which is also critically regulated by Ca^2+^ homeostasis [13]. In addition, Matsumoto et al. showed that cytosolic calcium regulates autophagy-related gene expression in autophagy-deficient mice [18] and that cytosolic Ca^2+^ signaling is a critical regulator of cardiac myocyte function [13]. A pivotal anti-senescence marker, SMP30 (senescence marker protein 30), has been reported to regulate intracellular Ca^2+^ homeostasis [19,20,21] and play a pathophysiological role in the tight regulation of Ca^2+^-related cell functions [22]. Recently, intracellular Ca^2+^ signaling has been reported to have an important role in the regulation of autophagy [23]; however, its exact function remains controversial.

The goal of this study was to clarify the molecular mechanism underlying the correlation between the autophagy-signaling pathway and DOXO-induced hCPC cytotoxicity, and to investigate whether the regulation of Ca^2+^-mediated autophagy signaling via mTOR signals restores or prevents DOXO-induced hCPCs cytotoxicity. The results indicate that DOXO-mediated hCPC cytotoxicity is closely related to Ca^2+^-related autophagy signaling and that inhibition of mTOR signaling by rapamycin may exert a cardio-protective effect against DOXO-mediated hCPC toxicity.

## 2. Results

### 2.1. Cytotoxicity Effect of Doxorubicin in hCPC

**Figure 1:**
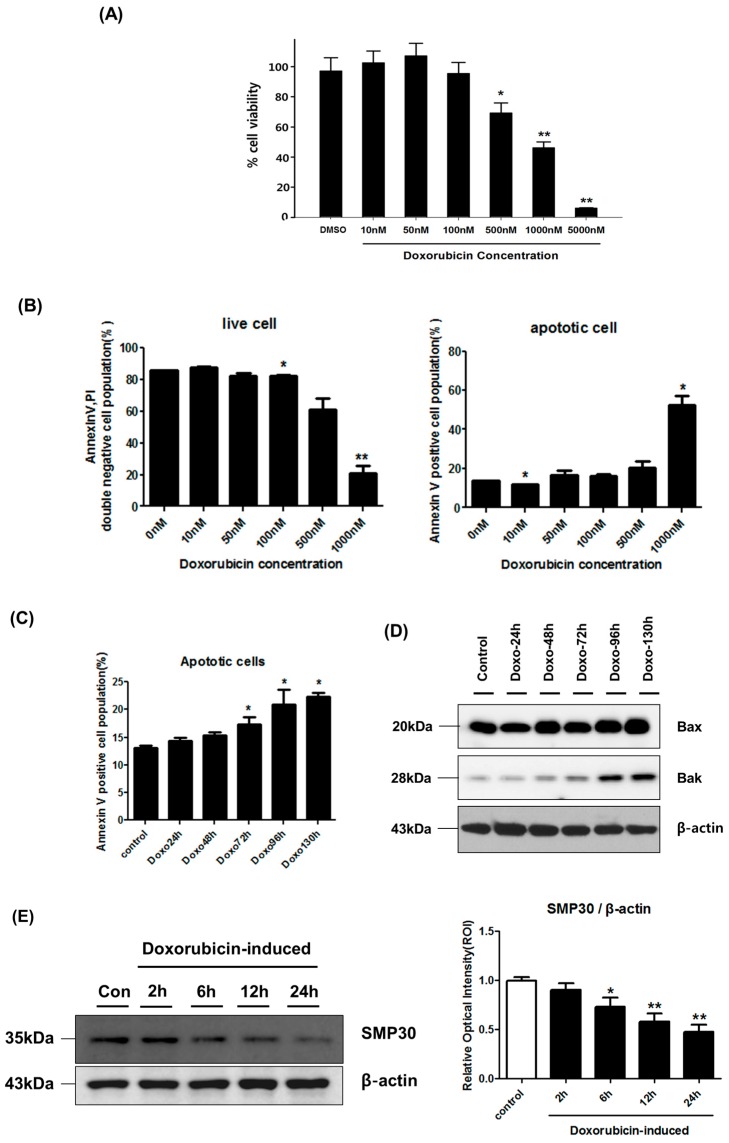
Effects of doxorubicin on hCPC cytotoxicity. (**A**) After treatment of hCPCs with doxorubicin (10, 50, 100, 500, 1000, 5000 nM) for 24 h, the cell viability was examined by MTS assay. * *p* < 0.05 versus DMSO control; ** *p* < 0.01 versus DMSO control; (**B**) hCPCs were treated with doxorubicin at various concentrations for 24 h, and the population of live and apoptotic cells were examined by Annexin V/PI assay. Live cells mean Annexin V/PI double negative cell population, while apoptotic cells mean annexin V positive cell population in this graph. * *p* < 0.05 versus 0nM control; ** *p* < 0.01 versus 0 nM (DMSO) control; (**C**) To determine doxorubicin-induced apoptosis in hCPCs, Annexin V/PI assay performed in a time-dependent manner. * *p* < 0.05 versus DMSO treatment control; (**D**) The expression of pro-apoptotic proteins including Bax and Bak was examined. hCPCs were treated with 100 nM doxorubicin for 130 h; (**E**) The expression level of SMP30, a senescence marker protein, was quantified by Western blot. * *p* < 0.05 versus control; ** *p* < 0.01 versus control. Data are mean ± SEM, n: total number; hCPCs: human cardiac progenitor cells.

We determined the effect of DOXO on hCPC cytotoxicity via the 3-(4,5-dimethylthiazol-2-yl)-5-(3-carboxymethoxyphenyl)-2-(4-sulfophenyl)-2*H*-tetrazolium (MTS) assay using serially diluted (10–5000 nM) DOXO for 24 h. As shown in Figure 1A, cellular viability decreased significantly at DOXO concentrations >500 nM (*p* < 0.05, Figure 1A). To address the cell apoptosis issue, we carried out Annexin V/PI assay at various doses of DOXO in hCPCs for 24 h. As shown in Figure 1B, a high dose of doxorubicin (1000 nM) significantly induced cell apoptosis, although it did not change at doses of 100 and 500 nM. Subsequently, we selected DOXO concentrations of 100 nM (chronic dosage) and 500 nM (acute dosage) to clarify our hypothesis. To further investigate DOXO-induced apoptosis in a time-dependent manner, we further examined the subpopulation of Annexin V/Propidium iodide (PI) at 100 nM treatment of DOXO, as well as the expression of pro-apoptotic proteins including Bax and Bak. As shown in Figure 1C,D, Annexin V(+) apoptotic cells significantly increased in a time-dependent manner and the expression of Bax and Bak also increased, suggesting that a high dose or long-term exposure to DOXO may induce cell apoptosis in hCPCs.

To elucidate that DOXO-mediated cytotoxicity may be related to Ca^2+^-mediated cellular dysfunction, we evaluated the expression of a Ca^2+^-mediated marker, SMP30 (also called regucalcin). As shown in Figure 1E, SMP30 decreased significantly in a time-dependent manner after treatment of hCPCs with DOXO for 24 h (*p* < 0.05), suggesting that in vitro treatment with DOXO leads to hCPC cell death via promotion of cellular dysfunction by reducing the expression level of the Ca^2+^-mediated marker, SMP30.

### 2.2. The Effect of Doxorubicin on Autophagosomes in hCPCs

**Figure 2:**
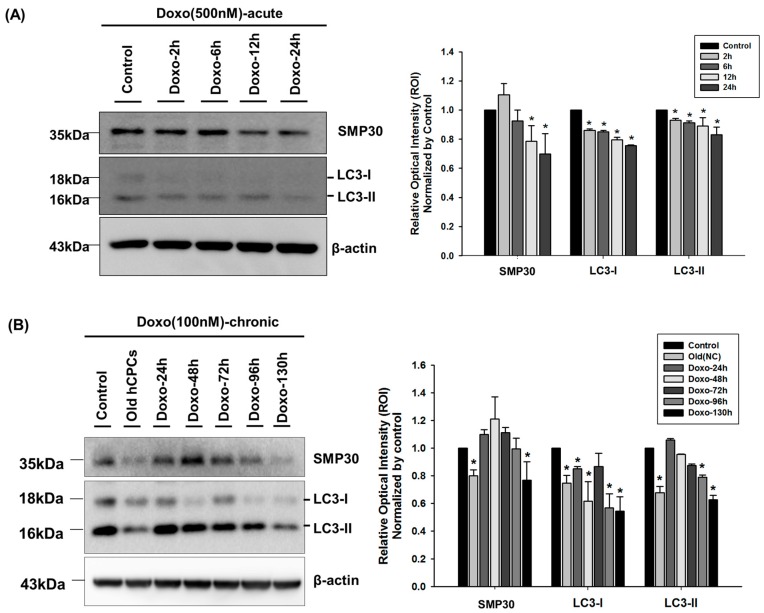

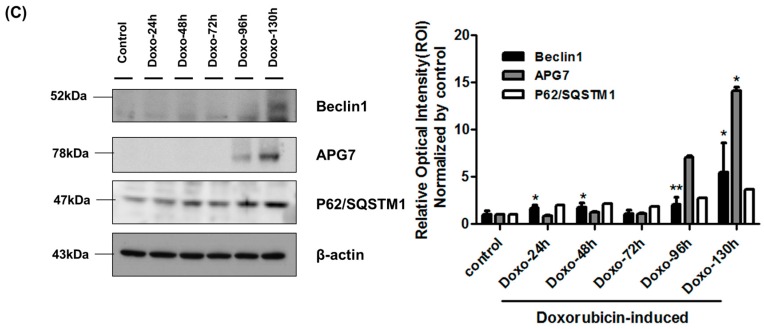
Effect of doxorubicin on expression of SMP30 and LC3. (**A**) The effect of 500 nM doxorubicin (acute dosage) on SMP30, LC3-I, and LC3-II protein expression at each time point, as detected by Western blot. The level of expression is shown in the bar graph on the right panel. * *p* < 0.05 versus control; (**B**) The effect of 100 nM doxorubicin (chronic dosage) on expression of SMP30, LC3-I, and LC3-II, as detected by Western blot. The level of expression was normalized to that of β-actin and is shown in the bar graph in the right panel. hCPCs at Passage 14 were used as senescence cells. * *p* < 0.05 versus control; (**C**) hCPCs were treated with 100 nM doxorubicin for 5 days, and we examined expression of autophagy-related proteins, Beclin-1, APG7 and P62/SQSTM1 by Western blot assay. * *p* < 0.05 versus DMSO treatment control; ** *p* < 0.01 versus DMSO treatment control.

A previous study reported that dysregulation of autophagy may contribute to DOXO- induced cardiotoxicity [14]. To evaluate the relationship between DOXO-mediated cytotoxicity in hCPCs and autophagy, we performed immunoblot assays using antibodies against an autophagosome marker, LC3, after exposure to doxorubicin for 24 h (acute dosage, 500 nM DOXO), 120 h (chronic dosage, 100 nM DOXO). As shown in Figure 2A, acute treatment with 500 nM DOXO significantly reduced SMP30 and LC3 expression. Similarly, chronic treatment of hCPCs with 100 nM DOXO significantly reduced the expression of SMP30 and LC3 in a time-dependent manner (Figure 2B), suggesting that DOXO-mediated cytotoxicity may be linked to autophagosome formation. 

To determine whether DOXO affects autophagy signaling, we examined other autophagy-related proteins, namely Beclin-1, APG7 and P62/SQSTM1. As shown in Figure 2C, the levels of these autophagy-related proteins significantly increased after doxorubicin treatment, suggesting that high dose or long-term exposure in DOXO might promote autophagy, eventually leading to cell apoptosis.

### 2.3. The Relationships between Doxorubicin and mTOR in hCPCs

**Figure 3:**
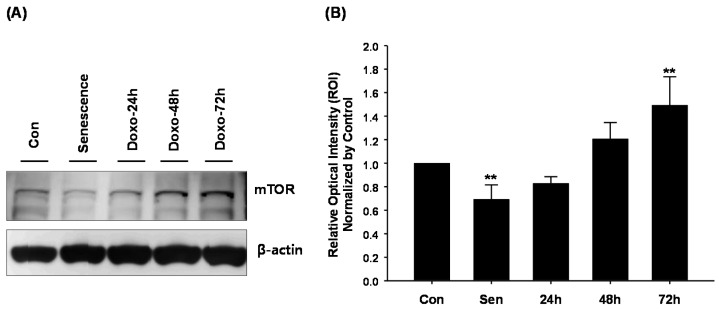

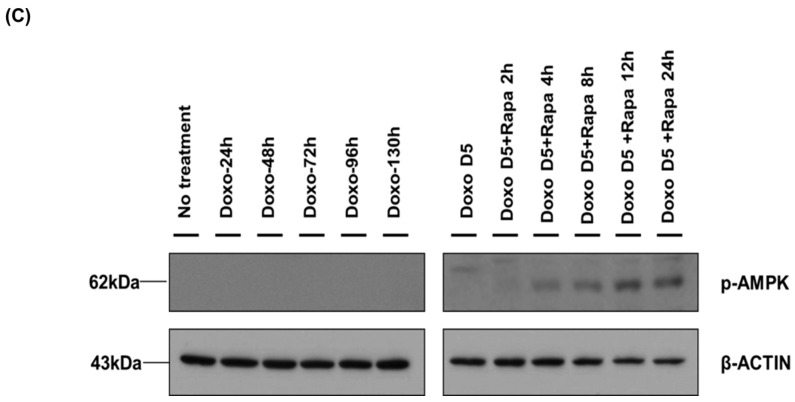
mTOR expression in doxorubicin-treated hCPCs. (**A**) mTOR expression by Western blot afterDOXO treatment for 24, 48 and 72 h. mTOR expression in hCPCs increased significantly in a time-dependent manner after treatment with DOXO. hCPCs at Passage 14 were used as senescence cells; (**B**) mTOR expression was quantified and normalized as per control. ** *p* < 0.01 versus control. Sen: Senescence hCPC; mTOR: mammalian target of rapamycin; (**C**) Increased AMPK phosphorylation in response to rapamycin-treated CPCs (at 100 nM DOXO for 5 days).

Accumulating evidence shows that autophagy is closely related to mTOR in mammalian cells. To clarify the relationship between autophagosome formation in hCPCs and mTOR signaling, we performed immunoblot assays using hCPCs after treating with 100 nM DOXO for 72 h. As shown in Figure 3A,B, mTOR expression was significantly increased, suggesting that DOXO-mediated autophagosome formation may be related to mTOR expression. To elucidate AMPK activity, a target mTOR, we evaluated phosphorylation of AMPK by Western blot. Doxorubicin-treated hCPCs did not affect AMPK phosphorylation. Interestingly, treatment of specific mTOR inhibitor, rapamycin, strongly induced AMPK phosphorylation (Figure 3C), suggesting that the mTOR-AMPK axis may be closely linked in DOXO-treated CPCs.

### 2.4. Restoration of DOXO-Mediated Autophagy Signaling and SMP30 by Rapamycin

**Figure 4:**
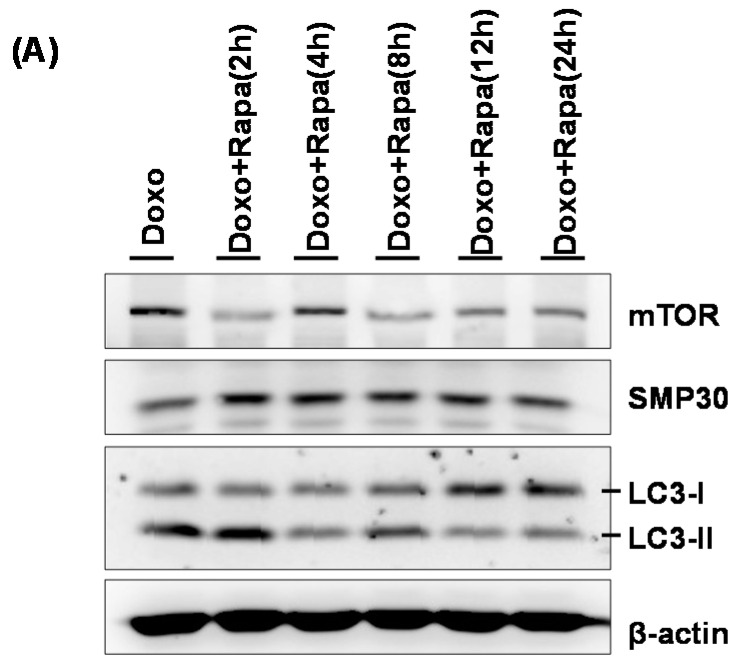

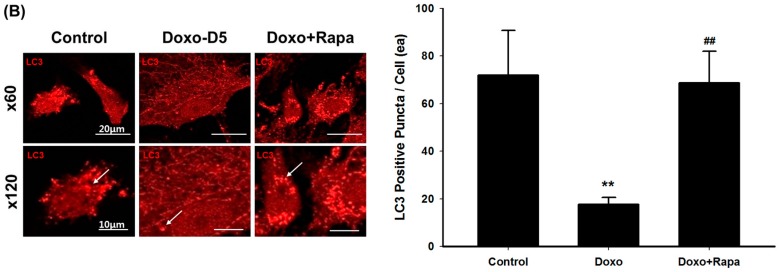
Rapamycin restores SMP30 and LC3 expression in DOXO-induced hCPCs. (**A**) hCPCs were incubated with doxorubicin (100 nM, 5 days) with or without stimulation by rapamycin (100 nM). The expression levels of mTOR, SMP30, and LC3 were assessed by Western blot. DOXO: doxorubicin, Rapa: rapamycin; DOXO + control: chronic treatment with doxorubicin hCPC (5 days); (**B**) Intracellular LC3 levels were measured using immunofluorescent assay and their fluorescence intensities were calculated by ImageJ software. Quantification of fluorescence intensities is presented in the right panel. ** *p* < 0.01 versus control, ## *p* < 0.01 versus doxorubicin. White arrows indicate LC3 autophagic puncta.

To confirm the previous findings that DOXO-mediated cytotoxicity in hCPCs leads to autophagy via regulation of mTOR signaling, we attempted to block mTOR signaling using rapamycin, a specific inhibitor, after treatment with rapamycin (100 nM) for 24 h. As shown in Figure 4A, we observed that treatment with rapamycin significantly restored the expression of LC3, an autophagosome marker, as well as re-expression of SMP30, a Ca^2+^-mediated anti-senescence marker. In addition, further autophagy puncta assay confirmed that inhibition of mTOR signaling by rapamycin in DOXO-treated hCPCs resulted in reduced autophagosome puncta numbers compared with control (Figure 4B), suggesting that clinically applicable rapamycin is a potent restoration factor against DOXO-induced hCPC toxicity via promotion of autophagosome formation.

### 2.5. Rapamycin Reduces DOXO-Mediated Cytosolic Ca^2+^ Accumulation in hCPCs

**Figure 5:**
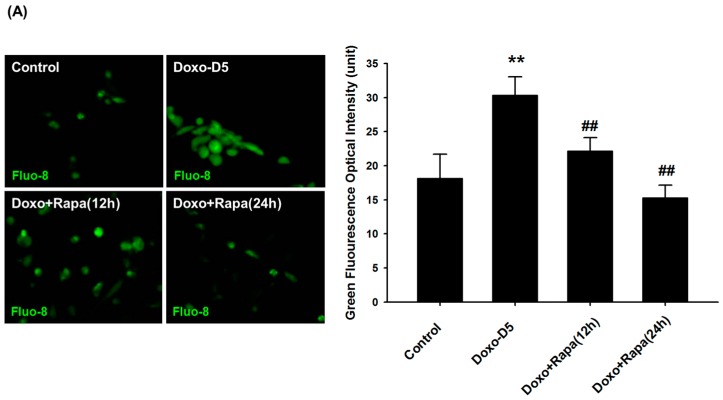

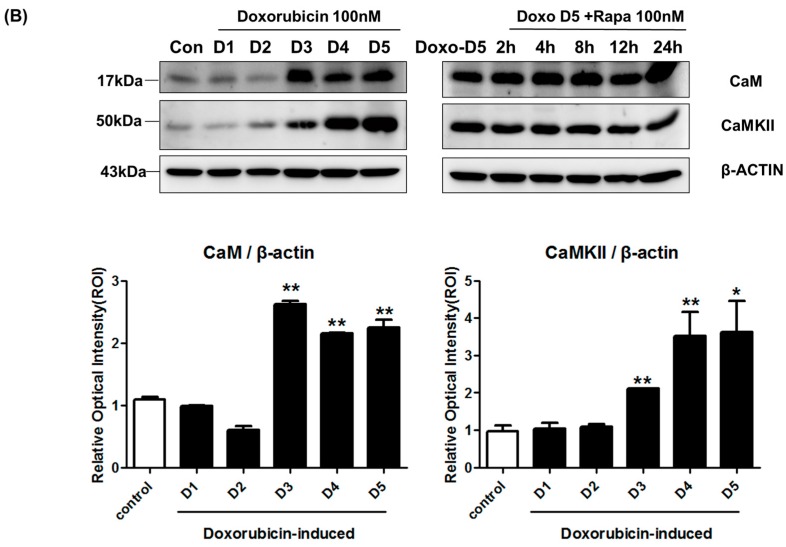
Rapamycin modulates cytosolic Ca^2+^ accumulation. (**A**) After treatment of hCPCs with doxorubicin (100 nM, 5 days) with rapamycin (100 nM, 12 and 24 h), intracellular Ca^2+^ levels were analyzed by Fluo-8 assay. The quantitative values of fluorescence intensity are presented. DOXO: doxorubicin, Rapa: rapamycin, ** *p* < 0.01 versus control; ## *p* < 0.01 versus DOXO-treated group; (**B**) After treatment of hCPCs with doxorubicin in a time-dependent manner (DOXO: 100 nM) and/or rapamycin (DOXO: 100 nM, 5 days), the expression of CaM and CaMKII was examined by Western blot analysis. * *p* < 0.05 versus control; ** *p* < 0.01 versus control.

To clarify our findings that Ca^2+^-mediated autophagy may be closely linked to DOXO-mediated hCPC cytotoxicity, we measured cytosolic Ca^2+^ levels using a chemical-specific dye in a Flou-8 assay. As shown in Figure 5A, cytosolic Ca^2+^ levels were significantly increased after treatment with DOXO. Interestingly, we found that treatment of rapamycin significantly reduced cytosolic calcium levels (*p* < 0.01, versus DOXO-treated group), suggesting that DOXO-mediated hCPC depletion may be associated with cytosolic Ca^2+^ accumulation.

We next evaluated the expression of calcium binding proteins, CaM (Calmodulin) and CaMKII (Calmodulin kinase II), in order to examine the relationship between Ca^2+^ accumulation and calcium-related signals. As shown in Figure 5B, we observed augmented expression levels of calcium storage proteins including CaM and CaMKII, although specific inhibition of mTOR by rapamycin did not change with the expression levels of CaM and CaMKII. This suggests that a distinct mechanism of action is exerted between mTOR-mediated Ca^2+^ accumulation and calcium storage proteins. Taken together, our data indicate that modulation of autophagy by rapamycin may prevent hCPC damage, in part, via modulation of Ca^2+^-mediated autophagy signaling.

## 3. Discussion

Autophagy is a catabolic process involving lysosomal degradation and recycling of protein aggregates that remove damaged organs to maintain normal cellular homeostasis [24,25]. Current literature on autophagy signaling shows that the onset of physiological autophagy plays a critical role in cell metabolism, as well as the intracellular signaling pathway for the regulation of cellular activities including cell proliferation, differentiation, and replicative senescence, including those of therapeutic stem cells [24,25,26]. In a clinical setting, CPCs are a promising source of cells for the treatment of ischemic cardiovascular diseases [10] as they contribute to the repair of damaged heart tissue via direct multi-lineage differentiation into various types of cells including ECs, CMs, and SMCs [11]. Although studies to date suggest that DOXO-induced cardiotoxicity leads to CSC depletion, the underlying mechanisms of DOXO-induced cardiotoxicity have not been fully determined.

In this study, we hypothesized that DOXO-mediated hCPC depletion may be associated with autophagy signaling and that the modulation of autophagy-related signals may potentially restore or prevent DOXO-mediated hCPC cytotoxicity. To first determine the relationship between DOXO-mediated CPC cytotoxicity and autophagy signals, we investigated expression levels of autophagosome formation markers, LC3-I and LC3-II. The results showed that expression of the autophagosome formation markers LC3-I and LC3-II was significantly reduced in DOXO-treated hCPCs. We also observed that expression of a calcium regulator SMP30 (called as regucalcin) was significantly reduced, indicating that Ca^2+^-mediated autophagy signaling may be associated with DOXO-mediated CPC depletion. Previous reports also suggest that reduced expression of SMP30 (regucalcin) indicates abnormal cytosolic Ca^2+^ handling and dysregulation of Ca^2+^-dependent enzymes in dystrophin-deficient muscles [18]. To understand the role of DOXO in hCPCs, we examined cytosolic Ca^2+^ levels and calcium-related molecules, CaM and CaMKII, and found that DOXO induces cytosolic Ca^2+^ accumulation and calcium-related regulators, suggesting that DOXO induces abnormal cytosolic Ca^2+^ handling. Of note is that recent studies have suggested that reduction of autophagosome formation leads to cellular accumulation of calcium-related damage to organelles to maintain normal cellular homeostasis [25,27]. Based on the results of the present study, we speculate that improper regulation and dysfunction of autophagy in DOXO-mediated CPCs may be the primary cause of CPC cytotoxicity.

The mTOR signal cascade has been reported to be closely linked to autophagy signaling [28,29,30]. To investigate DOXO-mediated autophagy signals, we investigated the expression of mTOR in DOXO-treated CPCs, and found that the mTOR expression significantly increased after 72 h of treatment with DOXO, suggesting that DOXO reduces autophagosome formation in hCPCs via increase in mTOR expression. These findings were confirmed by the finding that rapamycin, an inhibitor of mTOR, significantly recovered the autophagosome formation capacity of DOXO-treated hCPCs.

**Figure 6:**
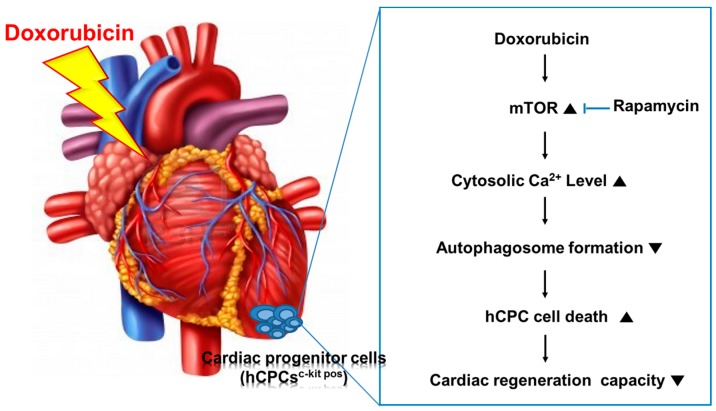
Schematic model of DOXO-related autophagy signaling in hCPCs; Rapamycin may exert a cardio-protective effect against DOXO-mediated cardiotoxicity and autophagy signaling via regulation of cytosolic Ca^2+^ accumulation.

Recently, it has been reported that dysregulation of autophagy might be closely related to an imbalance in cellular metabolism including mitochondrial dysfunction and cell toxicity [13,14,15]. Autophagy protects cells from stress stimuli and regulates cell homeostasis, including clearance of old organs, such as mitochondria and ER, while excess autophagy signals eventually lead to cell death [31]. Taken together, we first demonstrated that DOXO causes significantly abnormal cytosolic Ca^2+^ accumulation in human hCPCs, which disrupts normal mTOR-mediated autophagy signaling, and eventually leads to cellular senescence and cytotoxicity in hCPCs (Figure 6). In a clinical setting, the results of this study provide a pivotal clue suggesting that adequate modulation of both Ca^2+^ homeostasis and autophagy by the inhibition of mTOR signaling by rapamycin may exert a cardio-protective effect against DOXO-mediated cardiotoxicity.

## 4. Experimental Section

### 4.1. Isolation of C-kit^pos^ hCPC and Cultures

For this study, c-kit^pos^ hCPCs were isolated from infant human heart tissue as previously described [32]. The protocol was approved by the Ethical Review Board of Pusan National University Yangsan Hospital, Gyeongsangnam-do, Korea (IRB 05-2015-133). The study was conducted in accordance with the Declaration of Helsinki. Heart tissues were minced and incubated in 0.2% collagenase type II (Worthington Biochemical Corp., Lakewood, NJ, USA) solution at 37 °C for 30 min. Cardiac cells were washed with Ham’s F12 medium (Hyclone, Logan, UT, USA) containing 10% fetal bovine serum (FBS; Gibco, CA, USA) and 1× Penicillin streptomycin (PS; Welgene, Gyeongsan, Korea), and washed twice with phosphate-buffered saline (PBS), followed by centrifugation at 1500 rpm for 5 min. Single cardiac cells (Passage number 0; P0) were expanded at 37 °C in an hCPC expansion medium (Ham’s F12 medium containing with 10% FBS, 1X PS, 2.5 U human erythropoietin (hEPO; R&D systems, Minneapolis, MN, USA), 5 μg basic human recombinant fibroblast growth factor (bFGF; Peprotech, Rocky Hill, NJ, USA), and 0.2 mM glutathione (Sigma-Aldrich, St. Louis, MO, USA). When confluence reached 80%, hCPCc-kit^pos^ cells were obtained by magnetic-activated cell sorting using human c-kit-specific antibody (Miltenyi Biotech, Bergisch-Gladbach, Germany) and stored under liquid nitrogen until use.

### 4.2. Annexin V/PI Assays for Apoptosis

Annexin V/PI assays were carried out by flow cytometry according to the manufacturer’s protocol (BD Bioscience, San Diego, CA, USA). To examine the number of apoptotic cells, DOXO-treated hCPCs were washed twice with phosphate-buffered saline (PBS), and stained with annexin V-FITC and PI-PE. After staining for 15 min at room temperature in the dark, apoptotic cells were examined using flow cytometry (FACS Canto II, BD Bioscience, San Diego, CA, USA).

### 4.3.Cell Viability Assay 

The effect on hCPC viability (Figure 1) was measured by the MTS assay using Ez-cytox (Dail-lab, Seoul, Korea). Cells were seeded on 96-well plates in hCPC medium. After 12 h, the medium was replaced with hCPC medium containing serially diluted DOXO (Sigma-Aldrich). Plates were incubated at 37 °C, under 5% CO_2_. After 24 h, medium containing DOXO was replaced with MTS solution and plates were incubated for 2 h, after which the relative optical intensity was measured against a background control at wavelength of 450 nm at room temperature.

### 4.4. Immunoblot Analysis

Total proteins were isolated using RIPA buffer (Thermo Scientific, Rockford, IL, USA). After determining protein concentration, whole-cell lysates were separated by sodium dodecyl sulfate-polyacrylamide gel electrophoresis and transferred electrophoretically to polyvinylidene fluoride membranes (Millipore, MA, USA). The membranes were blocked with 5% skim milk in Tris-buffered saline with 0.1% Tween-20 (TBST) for 1 h at room temperature. The membranes were then overnight incubated at 4 °C with primary antibodies against human-specific anti-SMP30 (Santa Cruz Biotechnology, Santa Cruz, CA, USA) (Figure 2 and Figure 3), LC 3 (Abcam, Cambridge, MA, USA) (Figure 2 and Figure 3), mTOR (Abcam) (Figure 4), p-AMPK (Abcam) (Figure 3), Beclin-1 (Abcam) (Figure 2), APG7 (Abcam) (Figure 2), p62/SQSTM1 (Novus, MA , USA) (Figure 2), CaM (Santa Cruz Biotechnology) (Figure 4), CaMKII (Santa Cruz Biotechnology) (Figure 4) and β-actin (Santa Cruz Biotechnology) (Figure 2, Figure 3 and Figure 4). After incubation, the membranes were washed with TBST and incubated with peroxidase-conjugated secondary antibodies (Santa Cruz Biotechnology). The bands were visualized using a chemiluminescence solution (BioNote, Hwaseong, Korea) with the LAS-3000 imaging system (Fujifilm, Tokyo, Japan).

### 4.5. Autophagy Puncta Assay

hCPCs were seeded on a cover glass in 24-well culture plates and treated with DOXO and rapamycin for 24 h. Next, the plates were washed with PBS (pH 7.4) and fixed with 4% paraformaldehyde (Affymetrix, Santa Clara, CA, USA) at room temperature for 15 min. The plates were then washed with PBS and blocked using non-specific antibodies in 5% normal serum at room temperature for 1 h. Primary antibodies (RGN, 1:50 and LC3, 1:75) were added, and plates were incubated at 4 °C overnight. Subsequently, the primary antibodies were washed, and fluorescence dye-linked secondary antibodies (Alexa 488 and 594, Molecular Probes, Eugene, OR, USA) were added. The nuclei were stained with 4′-6-diamidino-2-phenylindole (Sigma-Aldrich), and cover glasses were mounted onto glass slides. Fluorescence (Figure 4) was observed with a confocal microscope (Olympus, FV-2000, Tokyo, Japan), and images were captured. Fluorescence intensity was calculated using Image J software (Free software form NIH, available on: http://rsb.info.nih.gov/ij/download.htm).

### 4.6. Intracellular Ca^2+^ Level Analysis

After treatment of hCPCs with DOXO and rapamycin, intracellular Ca^2+^ levels were measured (Figure 5) with Ca^2+^-staining chemical dyes using the Fluo-8 assay kit (Abcam). Fluo-8 staining was performed according to the manufacturer instructions. hCPCs were seeded on a cover glass in 24-well culture plates and treated with DOXO and rapamycin. Following the drug treatment, culture plates were washed twice with Hank's balanced salt solution (HBSS) for 5 min each. A Fluo-8 dye-loaded solution was then added at a concentration of 250 μL/well (20 μL Fluo-8 stock solution in 10 mL 1× assay buffer) and the plates were incubated for 30 min at room temperature. When the reaction was complete, the Fluo-8 dye-loaded solution was removed, and HBSS buffer was added. Images were then taken using a fluorescence microscope (Leica, DE/DM IRB, Wetzlar, Germany). Fluorescence intensity was calculated using Image J software (1.45 version, NIH, Bethesda, MD, USA).

### 4.7. Statistical Analysis

All experimental results are presented as the mean ± standard deviation (SD). Comparisons between the two groups were performed using the unpaired Student’s *t*-test. The data were considered statistically significant, if the corresponding *p*-values were < 0.05.

## 5. Conclusions

DOXO-mediated hCPC toxicity is linked to Ca^2+^-related autophagy signaling, and inhibition of mTOR signaling may provide a cardio-protective effect against DOXO-mediated hCPC toxicity.
